# Evaluation of the Microscanner C3 for Automated Cell Counting in Cerebrospinal Fluid Analysis

**DOI:** 10.3390/diagnostics14192224

**Published:** 2024-10-05

**Authors:** Insu Park, Minkyeong Choi, Eunji Lee, Seoyeon Park, Woong Sik Jang, Chae Seung Lim, Sun-Young Ko

**Affiliations:** 1 BK21 Graduate Program, Department of Biomedical Sciences, College of Medicine, Korea University, Seoul 02841, Republic of Korea; in2269@korea.ac.kr (I.P.);; 2 Departments of Laboratory Medicine, College of Medicine, Korea University Guro Hospital, Seoul 08308, Republic of Korea; 3 Departments of Emergency Medicine, College of Medicine, Korea University Guro Hospital, Seoul 08308, Republic of Korea; 4 Departments of Laboratory Medicine, College of Medicine, Korea University, Seoul 02841, Republic of Korea; 5 Departments of Laboratory Medicine, College of Medicine, Korea University Ansan Hospital, Ansan-si 15355, Republic of Korea

**Keywords:** CSF, cell count, hemocytometer, Neubauer chamber, flow cytometer, Microscanner

## Abstract

**Background**: Cerebrospinal fluid (CSF) analysis is essential for diagnosing various disorders affecting the central nervous system (CNS). Traditionally, CSF cell count analysis is performed manually using a Neubauer chamber hemocytometer, which is labor-intensive and prone to subjective interpretation. **Methods**: In this study, we evaluated the analytical and clinical performance of the Microscanner C3, an automated cell counting system, for CSF analysis using artificially prepared samples and 150 clinical CSF samples. **Results:** The lowest detectable white blood cell (WBC) count was 3.33 cells/µL, and the lowest detectable red blood cell (RBC) count was 3.67 cells/µL. The coefficients of variation (CV%) for the Microscanner C3 were lower than those for the Neubauer chamber at all cell concentrations. The correlation coefficients (R) between the Microscanner C3 and conventional methods were high: 0.9377 for WBCs and 0.9952 for RBCs when compared with the Neubauer chamber, and 0.8782 for WBCs and 0.9759 for RBCs when compared with the flow cytometer. Additionally, the Microscanner C3 showed good agreement with both the Neubauer chamber and flow cytometer in the Passing–Bablok regression analysis and Bland–Altman analysis for WBC count at all concentrations and RBC count at concentrations of 0–1000 cells/µL. **Conclusions**: The Microscanner C3 proved to be more sensitive, precise, and consistent compared to the conventional hemocytometer. The new system is also compact, convenient, and cost-effective, making it a valuable option for clinical laboratories.

## 1. Introduction

Cerebrospinal fluid (CSF) cell count analysis is an essential test for diagnosing infections, inflammations, autoimmune diseases, tumors, and other conditions that occur in the central nervous system (CNS). It also aids in evaluating the effectiveness of antibiotic or chemotherapy treatments and predicting prognosis [1,2]. Normal CSF samples contain minimal white blood cells (WBCs), typically fewer than 5/µL, except in newborns, where counts can reach up to 32/µL and no red blood cells (RBCs) [1]. The presence of blood cells in CSF is crucial for evaluating CNS-related disorders. An increased WBC count in CSF samples can indicate bacterial or viral meningitis, encephalitis, and other neurological disorders [1,2,3]. Additionally, an increase in RBCs can indicate post-traumatic subarachnoid hemorrhage, cerebral hemorrhage, or peripheral blood contamination during lumbar puncture [1,3,4].

CSF cell count analysis is traditionally performed manually via microscopic examination using a hemocytometer, particularly the Neubauer chamber, which is considered the gold standard in this context. This method has been widely adopted in clinical laboratories due to its low cost and rapid analysis capabilities. Despite its widespread use, the process is labor-intensive, requiring continuous staff training to maintain proficiency, which can be resource-consuming. Moreover, the subjective nature of microscopic interpretation leads to significant inter-examiner variability. Variation coefficients for hemocytometers were reported to be as high as 45%, and variability between examiners can range from 2.5% to 116% for WBCs (>300 × 10^6^/L) and up to 141% for RBCs (>300 × 10^6^/L) [5,6].

Several automated platforms have been developed to analyze CSF cell counts to address these limitations, with studies indicating their potential to replace the manual microscopic method in clinical laboratories [7,8,9]. Automated cell analyzers provide advantages such as increased efficiency and the capability to process larger volumes of samples. However, these systems necessitate regular calibration and maintenance, requiring skilled personnel for effective operation [7]. Additionally, the high initial investment and substantial physical footprint of these instruments may restrict their implementation in resource-limited settings [7,10]. Moreover, despite technological advancements, the lower threshold for reliable leukocyte counts remains approximately 20 cells/µL, which must be validated according to the specific device utilized [3,11]. Given that the critical range for clinical decision-making is between 5 and 30 cells/µL, this implies that cell counts below 30 cells/µL require manual verification. Furthermore, the lower limit for accurate erythrocyte detection using automated devices is approximately 1000 cells/µL [3,11]. Nevertheless, even low levels of erythrocytes may hold clinical significance. Consequently, despite the promising benefits of automation, Neubauer chamber-based microscopy remains the preferred method in many laboratories, striking a balance between cost, accessibility, and the need for reliable results.

To overcome the shortcomings of existing microscopic and flow cytometric methods, we have developed an automated cell counting system for CSF analysis using the Microscanner C3 (Biozentech, Seoul, Republic of Korea). The Microscanner C3 is a microchip-based automated image analysis device equipped with precision optics and image analysis software. This device is smaller and more affordable than a flow cytometer and can be operated by non-experts. The image analysis software can be used separately on another instrument if the image sizes match. In this study, we evaluated the analytical and clinical performance of the Microscanner C3 for CSF cell count analysis using artificially prepared and clinical CSF samples. A comparative evaluation of the Microscanner C3 was performed against existing methods using a Neubauer chamber and flow cytometry.

## 2. Materials and Methods

### 2.1. Sample Collection and Study Design

A total of 150 CSF samples were used in this study, obtained for diagnostic purposes from patients who visited Korea University Guro Hospital between November 2021 and February 2024. Samples not over seven days were collected as residual CSF specimens before disposal after the analysis and reporting of results were completed. For comparative evaluation between the Neubauer chamber, flow cytometer, and Microscanner C3 methods, appropriate residual samples with a volume exceeding 500 μL were selected and analyzed as quickly as possible. All samples were collected in sterile plain tubes via lumbar puncture without pretreatment. Since this study used residual samples scheduled for disposal, patient consent was not obtained. This study was approved by the Institutional Review Board of the hospital (IRB No. 2021GR0336).

### 2.2. Manufacturing Quality Control Materials for CSF Cell Counting

The analytical performance of the Microscanner C3 was evaluated in terms of linearity, limit of blank (LOB), limit of detection (LOD), and precision using artificially prepared WBC and RBC quality control materials [12,13]. WBCs from whole-blood samples were extracted using an RBC lysing solution. Briefly, 5 mL of OptiLyse C, No-wash Lysing Solution (Beckman Coulter, Brea, CA, USA), was added to 1 mL of whole blood and incubated for ten minutes. Then, 30 mL of phosphate-buffered saline (PBS, pH 7.4) was added, and the mixture was centrifuged at 2000× *g* for five minutes to separate the supernatant. After the supernatant was removed, the cells were resuspended in 1 mL of PBS to prepare the WBC cell suspension. This suspension was then mixed with cell-free CSF through serial dilution to generate WBC quality control materials. Cell-free CSF was prepared by collecting normal CSF samples (WBC: 0–5 cells/µL, RBC: 0 cells/µL). These CSF samples were centrifuged at 2000× *g* for five minutes, and the supernatant was filtered through a 0.2 µm syringe filter (Hyundai, Republic of Korea). Three replicate measurements using the Navios EX flow cytometer (Beckman Coulter, Brea, CA, USA) were performed to confirm the cell-free state. Whole-blood samples were used to prepare RBC quality control materials by mixing them with cell-free CSF via serial dilution. The concentrations of WBCs and RBCs in the quality control materials were determined as the average value of three replicate measurements using the Navios EX flow cytometer.

### 2.3. CSF Cell Counting via Microscanner C3

**Figure 1:**
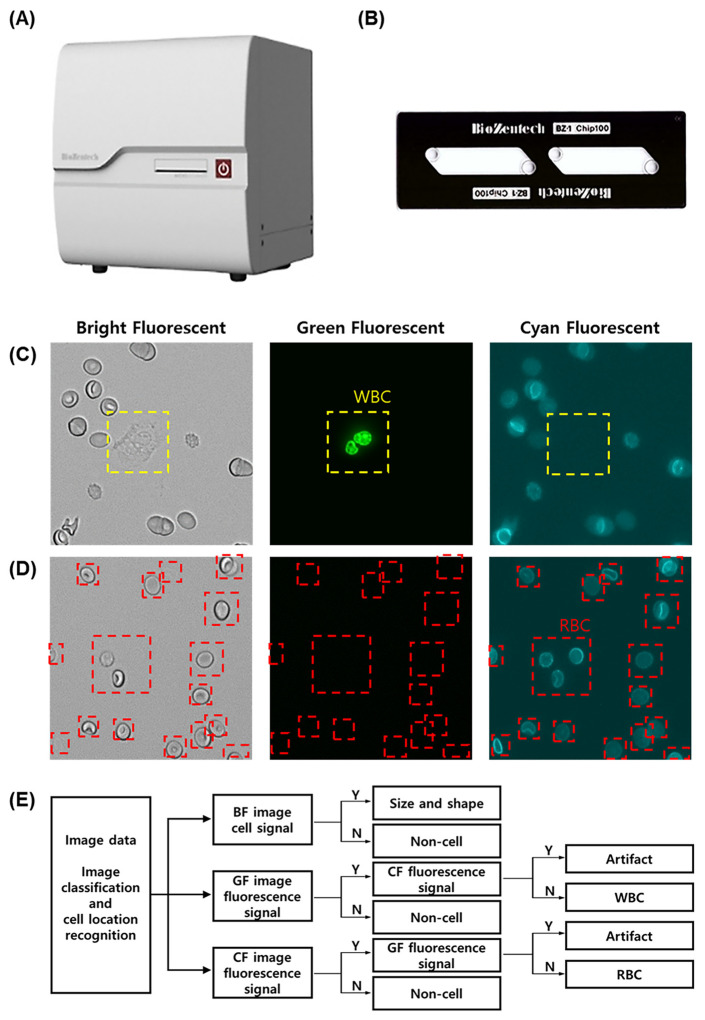
An overview of the Microscanner C3 for counting CSF cells. The Microscanner C3, equipped with a digital microscope and image analysis software, has dimensions of 245 mm (width) × 280 mm (depth) × 240 mm (height) and weighs 4.5 kg (**A**). The BZ-1 chip is loaded into the micro-scanner, and images are captured (**B**). The Microscanner C3 system captures single optical bright-field (BF), green-field (GF), and cyan-field (CF) images of blood cells in CSF. The images of BF, GF, and CF are obtained by photographing white blood cells stained with SYBR Green I (**C**) and by photographing red blood cells stained with APC mouse anti-human CD235a (**D**). The analysis workflow for the images describes the algorithm of the artificial intelligence-based hematology analysis program. WBCs and RBCs are classified based on the presence, location, size, and fluorescence of the cells within the three images (**E**).

The Microscanner C3 is a bench-top-sized instrument equipped with a ×40 magnification digital fluorescence microscope attached to a complementary metal oxide semiconductor camera (Figure 1A). Images can be acquired using an autofocus function that scans a disposable plastic microchip to a specific area that is automatically set and captures cells. The optical system consists of a bright field (BF) and three fluorescent light-emitting diode (LED) channels. In this study, BF images were obtained using green light (530 ± 20 nm) without an emission filter, green fluorescent (GF) images using blue light (470 ± 20 nm) and an emission filter (530 ± 25 nm), and cyan fluorescent (CF) images using red light (620 ± 30 nm) and an emission filter (700 ± 37.5 nm). The BZ-1 chip, a disposable plastic microchip used in this study, is made of polymethyl methacrylate optical material with dimensions of 25 mm (width) × 75 mm (depth) × 1.7 mm (height) and contains two microfluidic channels with a depth of 100 µm (Figure 1B). Each channel can hold a sample volume of 20 µL, and the loading time for each channel is approximately four seconds.

**Figure 2:**
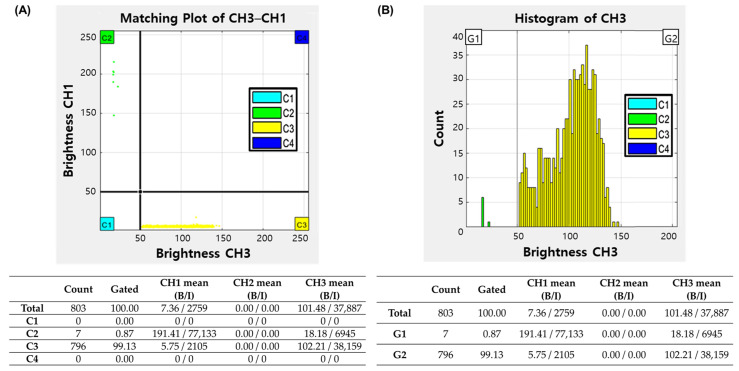
Results of the Microscanner C3 image analysis program. The X-axis represents the fluorescence intensity of the CF images, and the Y-axis represents that of the GF images. The graph is divided into four sections based on the staining strength of the cells (**A**). The C1 zone is defined as an area where both fluorescence signals are negative. Fluorescence signals in the C2 and C3 zones represent WBCs stained with SYBR Green I and RBCs stained with APC, respectively. The signals in the C4 area are differentiated using an artificial intelligence image analysis program to separate overlapping signals generated from WBCs, RBCs, or artifacts. The distribution of the two fluorescence signal intensities can be output as a histogram (**B**). Signals in WBCs are classified as the G1 zone (on the left), while signals in RBCs are classified as the G2 zone (on the right).

For image analysis, WBCs were labeled with SYBR Green I fluorescent dye (Invitrogen, Waltham, MA, USA) and RBCs with APC Mouse Anti-Human CD235a (Becton Dickinson, Franklin Lakes, NJ, USA). Additionally, the size range of the cells to be analyzed was fit on the instrument; thus, only WBCs and RBCs were analyzed specifically, and the influence of small-size air bulbs and dead cells in the fluid sample could be differentiated. A 20 μL aliquot of the mixture was injected into the BZ-1 chip, incubated at room temperature (RT) for three minutes in the dark, and then loaded onto the Microscanner C3. The instrument automatically photographed and analyzed sections of the microchip with auto-focusing (Figure 1C,D). The imaging time per sample was less than seven minutes. A circle Hough transform algorithm in MATLAB was employed to detect WBCs and RBCs in the CSF samples [13,14]. The images obtained from the Microscanner were analyzed using an artificial intelligence-based hematology analysis program. The algorithm was implemented using Python and the deep learning library PyTorch. The images captured by the Microscanner were transformed into multiple grid cells, allowing for the prediction of the presence, location, and size of objects using anchor boxes (Figure 1E). The automated clustering program performed CSF cell count analysis by enumerating the fluorescence signals and generating dot plots and histograms [15,16,17]. In the dot plots, the X-axis represents the intensity of the Cy5 channel, and the Y-axis represents the intensity of the FITC channel (Figure 2). The signals in the C2 zone were regarded as WBCs, and those in the C3 zone were RBCs. Signals in the C4 area, which are overlapping signals generated from WBCs, RBCs, or artifacts, were confirmed using an artificial intelligence cell image analysis program in the laboratory, and the number and percentage of signals were adjusted accordingly. The analysis time using both programs was less than five minutes. The following formula was used to calculate the WBC or RBC concentrations in the CSF samples:

WBC or RBC concentration in CSF (cells × 10^6^⁄L) = ((Total cell count × 1000))/((Volume per image captured by Microscanner C3 × Total number of images captured)) × Sample dilution factor × Dilution factor due to staining reagent

The dilution factor due to the staining reagent was calculated by dividing the total volume after staining (84 μL) by the CSF sample volume (40 μL), resulting in a dilution factor of 2.1. The volume per image captured by the Microscanner C3 was 23.8144 nL.

### 2.4. CSF Cell Counting via Neubauer Chamber

Analysis of WBC and RBC counts in CSF was performed using a Neubauer chamber (BLAUBRAND^®^, Wertheim, Germany) to compare results with those obtained using the Microscanner C3. An unstained CSF sample was pipetted onto one side of the chamber, filling 20 μL, and then examined under a ×400 magnification optical microscope. WBC counts were determined by counting cells in the four large grid squares at each corner of the chamber, while RBC counts were determined using the central grid square. To enhance result accuracy, five grid squares were counted when fewer than ten cells were present in each square. Each CSF sample was analyzed in duplicate, and the average of the two results is reported in cells × 10^6^/L.

### 2.5. CSF Cell Counting via Flow Cytometry

To evaluate the analytical and clinical performance of the Microscanner C3, we performed cell count analysis of the CSF samples using flow cytometry with a Navios EX flow cytometer (Beckman Coulter, Brea, CA, USA). For the preparation and analysis of WBC counts, the BD Leucocount kit (Becton Dickinson, Franklin Lakes, NJ, USA) was used according to the manufacturer’s instructions. Specifically, 100 μL of the CSF sample was mixed with 400 μL of Trucount reagent (Becton Dickinson, Franklin Lakes, NJ, USA) and incubated in the dark at room temperature (RT) for 15 min. Then, 500 μL of the stained sample was transferred to a BD Trucount™ tube (Becton Dickinson, Franklin Lakes, NJ, USA) and incubated in the dark at RT for an additional 15 min before analysis. For RBC count preparation and analysis, APC mouse anti-human CD235a monoclonal antibody (BD Pharmingen™, Franklin Lakes, NJ, USA) was used according to the manufacturer’s instructions. A total of 10 μL of the Anti-CD235a antibody, diluted 10-fold with PBS, was added to 100 μL of the CSF sample, mixed, and incubated in the dark at RT for five minutes.

For flow cytometry, 100 μL of the stained CSF sample was mixed with 100 μL of flow-count fluorospheres (Beckman Coulter, USA) in a flow cytometry tube. The CSF sample was analyzed for WBC and RBC counts using the Navios EX flow cytometer. WBCs were gated using side-scatter (SSC) and red fluorescence (FL3) dot plots, and cell numbers were calculated using absolute cell count formulas for beads and WBCs based on green and red fluorescence (FL1, FL3) dot plots. The formula used to determine the absolute count in the Trucount™ tube is as follows:

Absolute count of Trucount™ tube (cells × 10^6^⁄L) = ((Counted total cell number)/(Counted total bead number)) × ((Bead analysis concentration)/(Volume of tested sample)) × Sample dilution factor 

RBCs were gated using SSC and red fluorescence (FL6) dot plots, and absolute cell numbers were determined using bead counts identified over time and red fluorescence (FL5) dot plots. The formula for the absolute number of flow-count fluorospheres is as follows:

Absolute number of flow-count fluorophores (cells × 10^6^⁄L) = ((total number of cells counted)/(total number of beads counted)) × Bead assay concentration × Sample dilution factor 

Absolute cell counts were determined after acquiring more than 5000 events for each dot plot in accordance with the manufacturer’s instructions.

### 2.6. Performance Evaluation of Microscanner C3

The analytical performance of the Microscanner C3 for CSF cell count was evaluated following the guidelines of the Clinical & Laboratory Standards Institute (CLSI) and the International Council for Standardization in Hematology (ICSH) [18,19,20,21,22]. The evaluation involved tests for linearity, limit of blank (LOB), limit of detection (LOD), and precision using artificially prepared WBC and RBC quality control materials.

Linearity was assessed by analyzing WBC and RBC quality control materials at seven different concentrations ranging from 0.00 to 500.67 cells × 10^6^/L, measured in triplicate. The average values were used as the measured values. The Pearson correlation coefficient (r) was employed to assess the linear relationship between the measured and expected values for WBC and RBC counts.

LOB represents the maximum number of cells that can be measured in a blank sample. This was determined by performing 20 repeated measurements on two cell-free CSF samples over three days. LOB was defined as the 95th percentile of WBC and RBC counts in the cell-free CSF samples. LOD indicates the minimum number of cells that can be reliably detected as different from zero. LODs for the Microscanner C3 and Neubauer chamber were compared using the quality control materials for WBCs (0.66 to 7.00 cells × 10^6^/L) and RBCs (0.66 to 7.33 cells × 10^6^/L) across eight concentration ranges. Measurements were repeated 20 times for each sample, and LOD was defined as the minimum concentration value for which more than 95% of the measured values exceeded the LOB.

Precision was evaluated for the Neubauer chamber, Microscanner C3, and Navios EX flow cytometer using the WBC (4 to 2000 cells × 10^6^/L) and RBC (3.01 to 9400 cells × 10^6^/L) quality control materials across six concentration ranges. Each sample was measured six times over five days. The mean, standard deviation (SD), and coefficient of variation (CV%) were calculated.

The clinical performance of the Microscanner C3 was assessed through method comparison using 150 CSF samples from clinical patients. Each sample was analyzed twice using the Neubauer chamber, Microscanner C3, and flow cytometer. The WBC and RBC counts obtained from each method were compared, with the Navios EX flow cytometer values used as the reference standard. Pearson correlation coefficient (r), Passing–Bablok regression analysis, and Bland–Altman analysis were conducted. The Pearson correlation coefficient assessed the correlation between the methods while Passing–Bablok regression and Bland–Altman analyses were used to identify biases between the methods.

### 2.7. Statistical Analysis

Pearson correlation coefficient (r), Passing–Bablok regression analysis, and Bland–Altman analysis were performed. All statistical analyses were conducted using MedCalc^®^ statistical software version 9.3.2.0 (MedCalc, Mariakerke, Belgium) and Microsoft Office Excel^®^ 2019 (Microsoft, Redmond, WA, USA).

## 3. Results

### 3.1. Linearity

**Figure 3:**
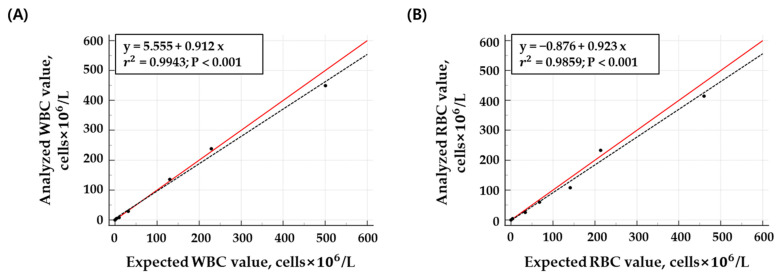
The linearity of cell counts was measured by the Microscanner C3 for WBCs (**A**) and RBCs (**B**) (*r*^2^ values: WBC = 0.9943, RBC = 0.9859). The agreement between the expected and measured cell counts is shown in a scatter plot with the line y = x (in red). The X-axis represents the expected cell counts of the quality control material, and the Y-axis represents the measured cell count results obtained from the Microscanner C3. Each concentration was measured three times.

The linearity of WBC counts (ranging from 0.00 to 500.67 cells × 10^6^/L) exhibited an *r*^2^ value of 0.9943, and for RBC counts (ranging from 0.00 to 460.33 cells × 10^6^/L), the *r*^2^ value was 0.9859, indicating a strong correlation (Table 1 and Figure 3).

### 3.2. Limit of Blank and Limit of Detection

The LOB for the Microscanner C3 was confirmed to be 0 cells × 10^6^/L for both WBC and RBC counts. The LOD values for the Neubauer chamber were 4.33 cells × 10^6^/L for WBCs and 4.33 cells × 10^6^/L for RBCs. In contrast, the LOD values for the Microscanner C3 were 3.33 cells × 10^6^/L for WBCs and 3.67 cells × 10^6^/L for RBCs (Table 2), indicating that the Microscanner C3 has lower LOD values compared to the Neubauer chamber.

### 3.3. Precision

The CV% for WBC counts ranged from 33.28% to 69.92% when analyzed with the Neubauer chamber, compared to 18.26% to 38.73% for the Microscanner C3. For RBC counts, the CV% ranged from 16.79% to 61.97% for the Neubauer chamber and from 6.71% to 34.99% for the Microscanner C3. In contrast, the CV% for WBC and RBC counts, when analyzed with the Navios EX flow cytometer, ranged from 2.8% to 18.07% and from 3.21% to 21.08%, respectively (Table 3). Thus, the Microscanner C3 demonstrated lower CV% values compared to the Neubauer chamber, indicating superior repeatability.

### 3.4. Clinical Sample Results

**Figure 4:**
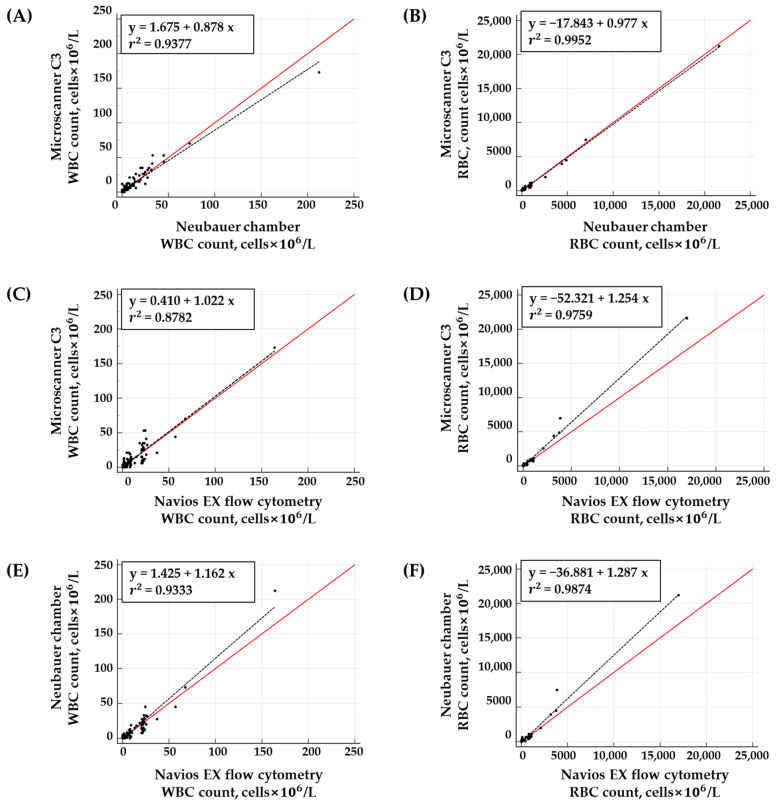
Correlation analysis plot of WBC (**A**,**C**,**E**) and RBC (**B**,**D**,**F**) counts in the CSF samples using the Neubauer chamber, Microscanner C3, and flow cytometer (N = 150). The baseline (y = x) is indicated by solid red lines, and the regression lines are indicated by dashed black lines.

The WBC count of the clinical CSF samples ranged from 0.0 to 164 cells × 10^6^/L, and the RBC count ranged from 0.0 to 17,000 cells × 10^6^/L when analyzed with the Navios EX flow cytometer. Strong correlations were observed between the results of WBC and RBC counts obtained from the three methods. The *r*^2^ values for the WBC and RBC counts between the Neubauer chamber and Microscanner C3 were 0.9377 and 0.9952, respectively. Between the Navios EX flow cytometer and Microscanner C3, the *r*^2^ values were 0.8782 and 0.9759, respectively. The *r*^2^ values between the Navios EX flow cytometer and Neubauer chamber were 0.9333 and 0.9874, respectively (Table 4 and Figure 4).

**Figure 5:**
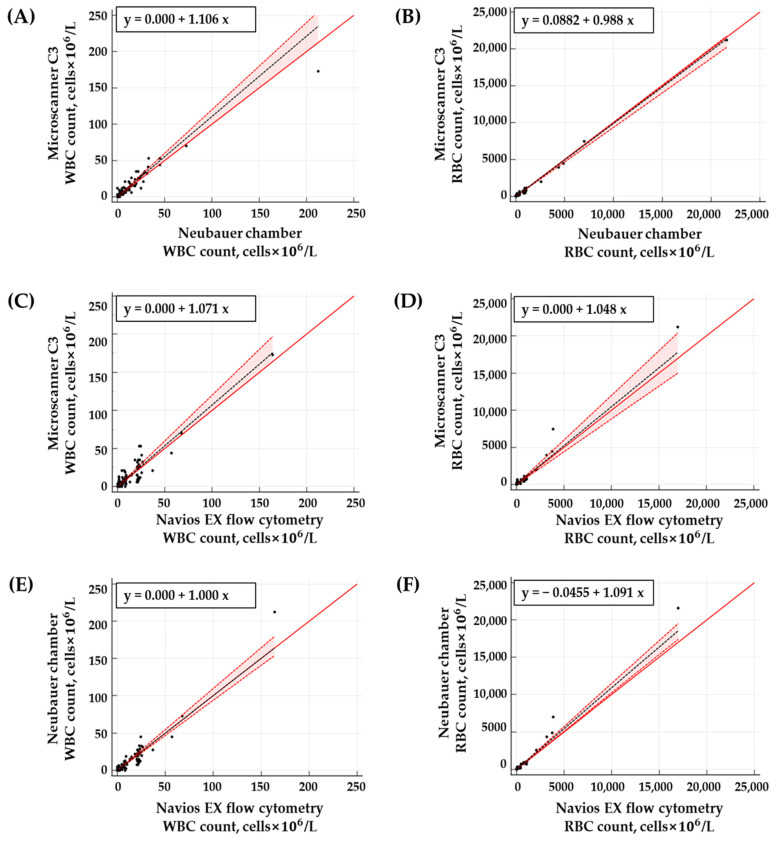
Passing–Bablok regression analysis of WBC (**A**,**C**,**E**) and RBC (**B**,**D**,**F**) counts in the CSF samples using the Neubauer chamber, Microscanner C3, and flow cytometer (N = 150). The baseline (y = x) is indicated by red solid lines, the slope by black dashed lines, and the 95% confidence interval by red dotted lines.

**Figure 6:**
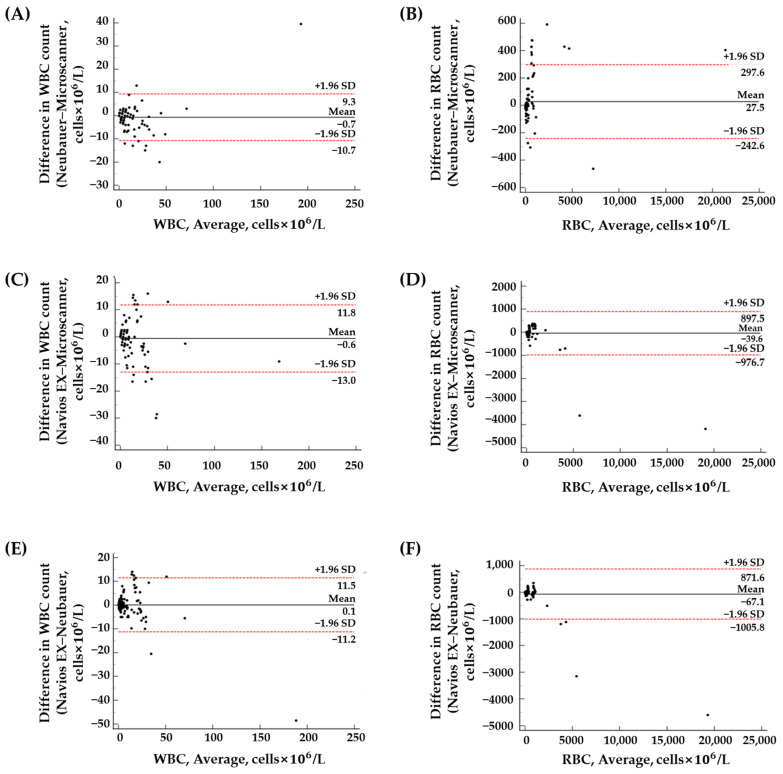
Bland–Altman analysis of WBC (**A**,**C**,**E**) and RBC (**B**,**D**,**F**) counts in the CSF samples using the Neubauer chamber, Microscanner C3, and flow cytometer (N = 150). The mean difference is indicated by horizontal black solid lines, while the limits of agreement, defined as the mean difference plus or minus 1.96 times the standard deviation of the difference, are indicated by red dotted lines.

The Passing–Bablok regression analysis revealed no significant differences in WBC and RBC counts between the Neubauer chamber and Microscanner C3 (Figure 5). However, the Bland–Altman analysis showed a mean bias of 27.52 (95% CI = 5.29 to 49.75) for RBC counts between these two methods (Figure 6). Nevertheless, within the RBC count range of 0 to 1000 cells × 10^6^/L (n = 140), the mean bias was 17.03 (95% CI = −1.49 to 35.54), indicating better agreement. No significant differences were found for WBC and RBC counts between the Navios EX flow cytometer and Microscanner C3 using both statistical analyses (Figure 4 and Figure 5). The Passing–Bablok regression analysis of cell counts between the Navios EX flow cytometer and Neubauer chamber confirmed a proportional bias for RBC counts (slope = 1.09, 95% CI = 1.02 to 1.15), and the Bland–Altman analysis showed no significant differences (Figure 5).

Overall, the Microscanner C3 demonstrated good agreement with the Neubauer chamber for the analysis of WBC and RBC counts in the CSF samples. Additionally, the Microscanner C3 produced consistent results with minimal bias, showing stability equivalent to or better than the Neubauer chamber for WBC counts up to 164 cells × 10^6^/L and RBC counts up to 1000 cells × 10^6^/L.

## 4. Discussion

The Neubauer chamber is a traditional gold standard for CSF cell count analysis. It is inexpensive and delivers rapid results, but it involves manual procedures that are labor-intensive and prone to subjective interpretation. In contrast, flow cytometry-based automatic hematology analyzers offer high precision and consistency. However, these instruments, such as the Navios EX flow cytometer used in this study, are large with dimensions of 953 mm (width) × 726 mm (depth) × 605 (height) and weigh 104 kg. They are also costly, with a price tag of approximately USD 100,000, and require specialized personnel for their maintenance and operation.

Various groups are developing image-based platforms for counting cells in CSF, with the primary challenge being how to achieve accurate and precise results, especially at low cell concentrations [23,24]. Accurate enumeration of WBCs and RBCs in CSF requires minimizing discrepancies between cell counts obtained from Microscanner C3 images and those from automatic image analysis systems. Several factors contribute to these discrepancies, such as cell-free debris, fluorescent aggregates, and overlapping cells. In unstained CSF samples mixed with PBS, cell-free debris and fluorescent antibody aggregates can appear in various shapes and sizes, complicating the analysis. Our laboratory’s AI image analysis program effectively identifies and removes these artifacts, improving accuracy. By recognizing these artifacts and distinguishing them from cells, we can reduce the discrepancies in cell counts. Enhancing AI programs with more diverse artifact images could further reduce errors. Moreover, cells that overlap in images may be counted as a single cell, leading to an underestimate of the total count. To address this, algorithms need to differentiate between single and overlapping cells based on their size and shape [24]. Taking more images can improve accuracy by providing a more comprehensive dataset. However, this approach can also increase analysis time.

This study assessed the analytical performance and clinical effectiveness of the Microscanner C3 and automatic image analysis device for CSF cell counting, comparing it to a conventional Neubauer chamber and flow cytometry. The Microscanner C3 demonstrated high linearity and low LODs for WBCs and RBCs. The Microscanner C3 showed a lower CV% compared to the Neubauer chamber across all cell count concentrations. Typically, the CV% for the Neubauer chamber was higher than the values reported in other studies, which is approximately 45% at five cells × 10^6^/L, likely due to instability from factors such as RBC lysis, cell aggregation, and WBC deformation during specimen storage and analysis. Overall, the Microscanner C3 provided more reliable and precise results for CSF cell counts, particularly at concentrations below five cells × 10^6^6/L, compared to the manual Neubauer chamber method [25].

In the comparative evaluation involving 150 clinical CSF samples, all three methods demonstrated a high degree of correlation with *r*^2^ values between 0.8782 and 0.9952 for WBCs and RBCs. The Passing–Bablok regression and Bland–Altman analysis indicated generally good agreement between the methods, with some exceptions in RBC counts. In the Bland–Altman analysis between the Neubauer chamber and Microscanner C3, a mean bias of 27.52 cells (95% CI = 5.29 to 49.75) was present. In the range of 0 to 1000 cells × 10^6^/L (n = 140), the mean bias was reduced to 17.03 (95% CI = −1.49 to 35.54). In the Passing–Bablok regression analysis between the flow cytometer and Neubauer chamber, the slope was 1.09 (95% CI = 1.02 to 1.15). The slope was 1.08 (95% CI = 1.02 to 1.13) in the range of 0 to 1000 cells × 10^6^/L (n = 140), indicating a consistent bias. In contrast, the slope value when comparing the flow cytometer and Microscanner C3 in the range of 0 to 1000 cells × 10^6^/L (n = 140) was 1.04 (95% CI = 0.86 to 1.27), demonstrating no significant difference. Overall, the Microscanner C3 exhibited good agreement with flow cytometry, performing comparably to or better than the Neubauer chamber (WBC range: 0 to 164 cells × 10^6^/L; RBC range: 0 to 1000 cells × 10^6^/L).

This study advocates for the use of the Microscanner C3 as a cost-effective and accessible solution for reliable CSF cell counting in resource-limited settings. The Microscanner C3 is compact and lightweight, with dimensions of 245 mm (width) × 280 mm (depth) × 240 (height) and a weight of 4.5 kg. It is also affordable, with an approximate cost of USD 20,000, and is capable of simultaneously detecting WBCs and RBCs using a small sample volume of 40 µL of CSF. This combination of affordability, compact size, and efficient sample utilization makes the Microscanner C3 advantageous in terms of both economic and spatial considerations. The Microscanner C3 is a versatile tool with potential applications across various diagnostic fields. In addition to its use for CSF cell count analysis, clinical research has demonstrated its effectiveness with a range of clinical samples. Previous studies have highlighted its potential in several areas, including malaria detection, transfusion medicine, and immunophenotyping [26,27,28]. This suggests that the Microscanner C3 could be broadly applicable in diverse diagnostic contexts.

## 5. Conclusions

An automated cell counting system for CSF analysis using the Microscanner C3 was developed and evaluated in this study, which proved to be more sensitive and precise compared to using a Neubauer chamber. The correlations between the results obtained using the Microscanner C3 and those using the Neubauer chamber and flow cytometer were high, showing good agreement with both methods for analyses of WBC and RBC counts in the CSF samples. In addition, the Microscanner C3 appeared to produce consistent results without a large increase or decrease bias, equivalent to or more stable than the Neubauer chamber. The new system is also compact, convenient, and cost-effective compared to the flow cytometer, making it a useful option for analyzing CSF.

## Figures and Tables

**Table 1:** Linearity of WBC and RBC concentration calculations using WBC and RBC quality control materials, analyzed using the Microscanner C3 (*n* = 3).

WBC (Cells × 10^6^/L)		RBC (Cells × 10^6^/L)	
Expected Value	Analyzed Mean ± SD	Expected Value	Analyzed Mean ± SD
0.00	0 ± 0.0	0.00	0. ± 0.0
3.33	4 ± 1.7	3.67	4 ± 1.7
9.67	8 ± 1.7	34.00	25.33 ± 4.0
31.33	29 ± 5.2	68.33	59.67 ± 22.7
130.33	136 ± 6.9	141.00	107.33 ± 12.6
229.33	238 ± 9.0	213.67	233.33 ± 22.9
500.67	449 ± 85.7	460.33	414.33 ± 24.0

**Table 2:** LOD values of the Neubauer chamber and Microscanner C3 for WBC and RBC counts using different concentrations of WBC and RBC quality control materials (N = 20).

Neubauer Chamber			Microscanner C3		
WBC (Cells × 10^6^/L)	Mean ± SD	CV%	WBC (Cells × 10^6^/L)	Mean ± SD	CV%
7	7.0 ± 4.3	60.82	7	6.9 ± 2.1	30.40
5.33	4.9 ± 3.2	66.32	5.33	5.6 ± 1.9	34.19
4.33	4.5 ± 3.1	69.29	4.33	4.2 ± 1.5	34.50
3.33	1.5 ± 1.7	113.43	3.33	3.8 ± 1.3	35.54
2.67	1.1 ± 1.5	134.40	2.67	1.5 ± 1.8	121.40
1.67	0.6 ± 1.4	220.05	1.67	0.9 ± 1.7	190.41
1	0 ± 0	-	1	0 ± 0	-
0.66	0 ± 0	-	0.66	0 ± 0	-
**RBC (cells** **× 10^6^/L)**	**Mean ± SD**	**CV%**	**RBC (cells** **× 10^6^/L)**	**Mean ± SD**	**CV%**
7.33	6.6 ± 2.6	38.99	7.33	7.1 ± 2.1	31.40
6	5.5 ± 2.3	41.28	6	6.2 ± 2.0	32.92
5	4.5 ± 2.1	46.85	5	4.5 ± 1.5	34.43
4.33	3.9 ± 2.6	67.53	4.33	4.2 ± 1.5	35.90
3.67	2.6 ± 1.9	71.03	3.67	3.9 ± 1.4	36.17
2.33	0.3 ± 1.0	326.24	2.33	0.9 ± 1.4	156.72
1.33	0 ± 0	-	1.33	0 ± 0	-
0.66	0 ± 0	-	0.66	0 ± 0	-

**Table 3:** The precision of the Neubauer chamber, Microscanner C3, and flow cytometer for WBC and RBC counts using six different concentrations of WBC and RBC quality control materials (N = 6).

	WBC (Cells × 10^6^/L)	4	8	20	100	500	2000
Neubauer chamber	Mean ± SD	3.6 ± 2.6	7.5 ± 4.5	17.9 ± 10.1	83.3 ± 40.8	516 ± 171.75	1833.3 ± 752.8
	CV%	69.92	59.63	56.1	48.99	33.28	41.06
Microscanner C3	Mean ± SD	4.0 ± 1.6	6.5 ± 2.3	16.0 ± 5.3	73.5 ± 15.5	421.2 ± 95	1800.0 ± 328.6
	CV%	38.73	34.74	32.83	21.09	22.56	18.26
Flow cytometer	Mean ± SD	4.2 ± 0.8	8.7 ± 0.8	17.2 ± 1.5	91.5 ± 6.4	502.4 ± 30.7	1951.8 ± 54.7
	CV%	18.07	9.42	8.57	6.94	6.19	2.8
	**RBC (cells × 10^6^/L)**	**3.01**	**15.04**	**75.2**	**376**	**1880**	**9400**
Neubauer chamber	Mean ± SD	3.3 ± 2.1	12.7 ± 4.1	101.7 ± 29.9	468.3 ± 90.2	2500.0 ± 429.0	13,666.7 ± 2294.9
	CV%	61.97	32.61	29.45	19.26	17.16	16.79
Microscanner C3	Mean ± SD	3.5 ± 1.2	17.5 ± 4.0	70.5 ± 9.6	371.3 ± 41.1	1958.3 ± 196.2	8520.0 ± 571.8
	CV%	34.99	22.79	13.57	11.08	10.02	6.71
Flow cytometer	Mean ± SD	3.0 ± 0.6	12.2 ± 1.5	64.2 ± 5.8	367.2 ± 25.4	1988.3 ± 128.7	9827.7 ± 315.2
	CV%	21.08	12.1	9.02	6.91	6.47	3.21

**Table 4:** Comparison of the Neubauer chamber (NC), Microscanner C3 (MS), and flow cytometer (FC) for CSF cell counts using 150 clinical samples.

Method	Cell	*r*^2^ Value	Passing–Bablok Regression		Bland–Altman Plot	
			Slope (95% CI)	Intercept (95% CI)	Mean Bias (95% CI)	Mean Bias ± 1.96 SD
NC vs. MS	WBC	0.9377	1.11 (1.00~1.20)	0.00 (0.00~0.00)	−0.71 (−1.53~0.12)	−10.73~9.32
	RBC	0.9952	0.99 (0.94~1.01)	0.09 (0.00~1.23)	27.52 (5.29~49.75)	−242.56~297.59
FC vs. MS	WBC	0.8782	1.07 (1.00~1.20)	0.00 (0.00~0.00)	−0.59 (−1.61~0.43)	−12.95~11.77
	RBC	0.9759	1.05 (0.88~1.20)	0.00 (−0.30~1.01)	−39.61 (−116.74~37.53)	−976.70~897.48
NC vs. FC	WBC	0.9333	1.00 (0.94~1.10)	0.00 (0.00~0.00)	0.12 (−0.8199~1.05)	−11.24~11.47
	RBC	0.9874	1.09 (1.02~1.15)	−0.05 (−0.45~0.00)	−67.12 (−144.39~10.15)	−1005.81~871.57

## Data Availability

The authors declare that all related data are available from the corresponding author upon reasonable request.
